# Highly Soft, Abrasion-Resistant, and Moisture-Absorbent Wool/PA56 Blended Yarns for Seating Fabrics

**DOI:** 10.3390/polym16142052

**Published:** 2024-07-18

**Authors:** Shuangquan Wu, Zebo Wang, Xinhou Wang, Jinhua Jiang

**Affiliations:** 1 Key Laboratory of Textile Science and Technology, Ministry of Education, College of Textiles, Donghua University, Shanghai 201620, China; 1209702@mail.dhu.edu.cn; 2 Kuangda Automotive Trim System Co., Ltd., Changzhou 213100, China; wangdzyb@163.com; 3 Engineering Research Center of Technical Textile, Ministry of Education, College of Textiles, Donghua University, Shanghai 201620, China

**Keywords:** knitting, seating fabrics, yarns, wool, PA56

## Abstract

Biobased nylon (PA56) not only has the same physical properties as nylon (PA6/PA66) but its production method is also more environmentally friendly. PA56 fabric has the advantages of moisture absorption, perspiration, high-temperature resistance, and flexibility, which have been widely studied by scientific researchers. Wool has the advantages of beauty, environmental protection, and anti-wrinkle. However, pure wool fabrics have low strength and are easy to shrink when washed, which has always been a problem. Hence, this work adopted the ring spinning method to prepare wool/PA56 blended yarn with wool content of 0, 10, 30, 50, 70, and 100 wt%. Thus, to examine the effects of different blending ratios and twists on yarn performance, PA56 was blended with wool. The results showed that findings indicate that yarn performance is influenced by both yarn twist and blending ratio. The yarn thickens and takes on more linear density as the blending ratio and yarn twist increase. As the wool ratio increases, the yarn’s breaking stress and breaking strain decrease. It is obvious that the strength and elongation at break of pure PA56 yarn are 2.09 cN/Dtex and 33.92%, respectively. When the wool content was 100 wt%, the strength and elongation at break of the blended yarn were 0.66 cN/Dtex and 21.15%, respectively. With the amount of wool blending, the yarn hairiness index’s H-value initially rises and subsequently falls. The percentage of blended wool reaches 50% at 2.14; less blending might exacerbate the yarn’s stem, resulting in neps and unevenness features. The quality of the yarn improves as the blending percentage rises. The yarn has the advantages of resource saving, biodegradability, and environmental friendliness and has a broad application prospect in the automotive interior field.

## 1. Introduction

Nylon fiber, also known as polyamide fiber, is the world’s first industrial production of synthetic fibers [1,2]. Nylon fibers have excellent properties, with the best abrasion resistance among synthetic fibers, high strength and low initial modulus, soft and tough fibers, high resilience, and some moisture absorption properties. Polyamide 6 (PA6) and polyamide 66 (PA66) are two representatives of polyamide fibers. PA6 is produced through ω-amino acid polycondensation or lactam ring-opening polymerization, and PA66 is produced through the polycondensation of diamines with dibasic acids. However, the raw materials of PA6 and PA66 basically come from non-renewable fossil fuels, and their production process involves a large amount of resource consumption and environmental pollution. They not only increase the environmental burden but also run counter to the direction of carbon-neutral development [3,4]. Therefore, environmentally friendly biobased PAs have received much attention as represented by polyamide 56 (PA56), a novel polyamide produced from biobased pentamethylene diamine and adipic acid via a condensation synthesis process [5,6,7], while the microbial fermentation of starch to obtain biobased pentamethylene diamine has lowered the cost of the production of PA56 [8]. PA56 is much greener in the production path [5,9]. Compared to PA66, biobased PA56 can reduce the consumption of non-renewable resources by about 50%. Unlike PA66, PA56 contains unsaturated hydrogen bonds, which retain biological “activity” and provide strong abrasion resistance while imparting a natural soft touch, excellent drapability, and biodegradability [10,11]. Studies have shown that PA56 has excellent properties that make it an excellent alternative to traditional petroleum-based nylons [12,13,14], with promising applications in medical, textile, food packaging, electronic equipment, and automotive parts [15,16]. The development and application of the new nylon PA56 can effectively promote the sustainable development of the polyamide industry and generate greater economic value and environmental benefits.

Wool is a biodegradable and biocompatible natural fiber, wool fibers have better elasticity and the natural curling of single fibers, and its elongation at break can reach more than 80%, which is the best among natural fibers [17]. Wool fiber has the advantages of a soft luster, a rich and elastic feel, better drape, wrinkle resistance, thickness, and warmth [18]. However, wool yarn is not strong enough [19] to make up for this shortcoming, many researchers have conducted a lot of research on such topics as PA6/wool blending, PA66/wool blending, polyester/wool blending, and so on [18,20,21,22]. However, the use of many synthetic fibers causes the waste of non-renewable resources such as oil on the one hand [23,24] and the non-decomposable properties cause great damage to the environment on the other hand [25].

In this work, a wool/PA56 blended yarn was prepared by blending biobased PA56 with wool fibers, and the effects of different blending ratios and twisting degrees on the performance of the yarn were investigated. The blended fibers prepared in this study are applied in the field of wool spinning, the addition of wool fibers can not only enhance the luster, elasticity, hand feel, and comfort properties of the blended fabrics but also make up for the phenomenon of the lack of wool strength. The yarn has the advantages of resource saving, biodegradability, and environmental friendliness and has a broad application prospect in the textile field.

## 2. Materials and Methods

### 2.1. Materials

**Figure 1:**
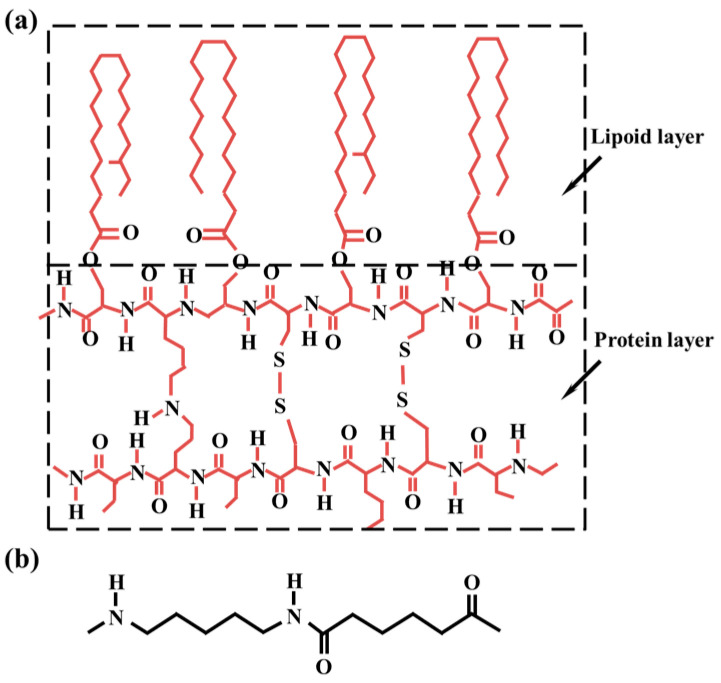
(**a**) Wool fiber structural formula; (**b**) PA56 structural formula.

The structural formula of wool fibers is shown in Figure 1a; wool fibers (70s merino wool) were purchased from (Zhejiang Xin Ao Textile Co., Ltd., Tongxiang, China). The structural formula of PA56 is shown in Figure 1b; PA56 staple fibers (molecular weight: 19,000, 2.2D × 51 mm, 2.2D × 64 mm, 2.2D × 76 mm, 2.2D × 88 mm) were purchased from (Suzhou Jinquan New Material Co., Ltd., Suzhou, China).

### 2.2. Preparation of Blended Yarns

**Figure 2:**
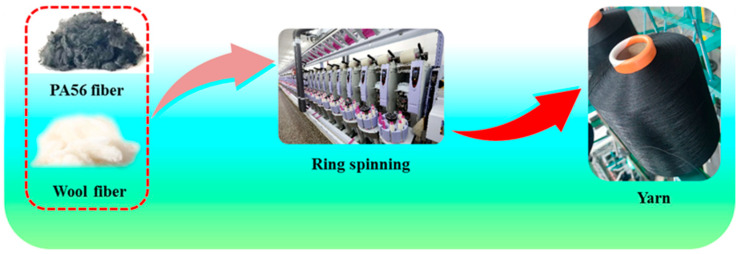
Preparation process of PA56/wool blended yarns.

Using the bulk fiber mixing method (weighing, manual layup, and mixing), the PA56 staple fiber and wool components were weighed and mixed according to the blending ratio. The blended yarn was made by a DSCa-01 digital sample carding machine, DSDr-01 digital sample drawing-in machine, DSR0-01 digital sample roving machine, and DSSp-01 digital sample spinning tester; the PA56/wool blended yarn was prepared as shown in Figure 2. PA56 staple fibers were electrostatically treated according to previous research results.

### 2.3. Measure Methods and Equipment

For determining the yarn’s linear density, the tests were executed with a YG086 Yarn Length Measuring Instrument (Changzhou No.1 Textile Equipment Co., Ltd., Changzhou, China) according to GB/T4743 [26] standards. 

The wicking performance test used an RF4008WV Vertical Wicking Tester (Shenzhen Ruifeng Instrument Co., Ltd., Shenzhen, China), tested according to the AATCC Test Method 197-2018 [27].

The boiling water shrinkage of the yarn was tested according to the GB/T6505 [28] test standard, using the YG089E Shrinkage Tester (A1/A2 Type) instrument (Wuhan Guoliang Instrument Co., Ltd., Wuhan, China).

Morphologies and microstructures of blended yarn were observed using a scanning electron microscope (SEM Quanta 250 FEG).

The strength and elongation at break of yarn test was carried out according to GB/T3916-2013 [29] test standard, using a YG023B-III Fully Automatic Single Yarn Strength Tester instrument (Changzhou No.1 Textile Equipment Co., Ltd., Changzhou, China), and the test speed was 1000 mm/min.

The analysis of yarn evenness and unevenness was carried out according to the GB/T 3292.2-2009 [30] standard using the Uster Evenness Tester 6-C800 (Wuhan Guoliang Instrument Co., Ltd., Wuhan, China).

The analysis of yarn hairiness was carried out according to the FZ/T10186-2000 [31] standard and the Yarn Hairiness Tester (Wuhan Guoliang Instrument Co., Ltd., Wuhan, China) was used for testing and analysis.

The wear resistance of the yarn was tested using the friction and wear test method [32], using an AT500 Yarn Abrasion Tester instrument (Shanghai Bingjing Instrument Equipment Co., Ltd., Shanghai, China), with a pre-tension of 30 cn, an unwinding speed of 100 m/min, and a test time of 300 s.

## 3. Results and Discussion

### 3.1. Linear Density Analysis of Yarns

**Figure 3:**
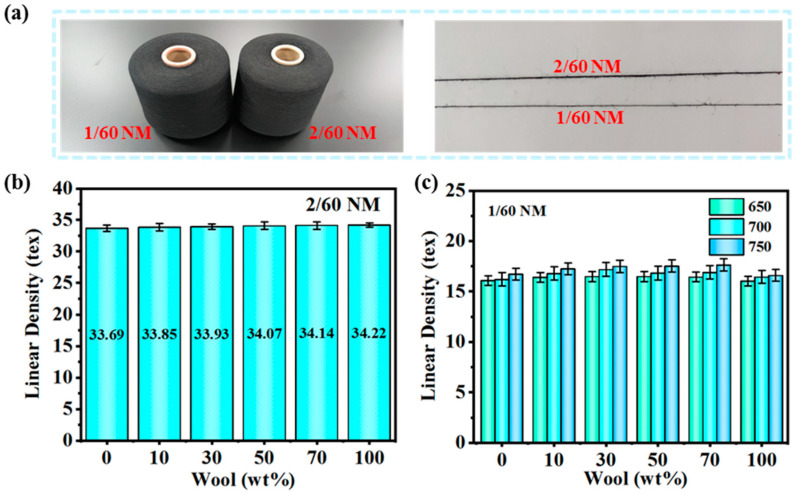
Thread densities of blended yarns with different wool contents: (**a**) 1/60 NM yarn and 2/60 NM yarn pictures, (**b**) 2/60 NM yarn, and (**c**) 1/60 NM yarn blended at 650, 700, and 750 T/m.

Illustrated in Figure 3 are the line densities for different wool contents of 0, 10, 30, 50, 70, and 100 wt%. From Figure 3a, it can be clearly seen that the 2/60 NM yarn is thicker than the 1/60 NM yarn. Figure 3b shows the line density of the 2/60 NM wool and PA56 blended yarns and Figure 3c shows the line density of 1/60 NM and twisted 650, 700, and 750 T/m wool/PA56 blended yarns. The density of wool is 1.25 g/cm^3^ and the density of PA56 is 1.14 g/cm^3^. There is not much difference between the two densities. With the increase in wool content, the change in the linear density of wool/PA56 blended yarn increases slowly with the increase in wool content, as shown in Figure 3. For the 1/60 NM yarn shown in Figure 3c, the thread density of the yarn increases as the twist increases. The reason for this is that the increase in yarn twist increases the yarn content per unit length, resulting in an increase in the thread density of the blended yarn. The reason for this is that during twisting, the spaces between the fibers decrease and the fibers become denser. This means that more fibers are wrapped together within the same length, increasing the density of the yarn. As the twist increases, the frictional resistance between the fibers also increases. This increased frictional resistance allows the fibers to be more tightly positioned in the yarn and less likely to slip, thus increasing the density of the yarn. The thread density of pure PA56 yarn with a 2/60 NM twist of 700 T/m is 33.69 tex and the thread density of pure wool is 34.22 tex, the thread density of pure PA56 yarn with a 1/60 NM twist of 700 T/m is 16.20 tex and the thread density of pure wool is 16.44 tex.

### 3.2. Wicking Height Analysis of Yarns

**Figure 4:**
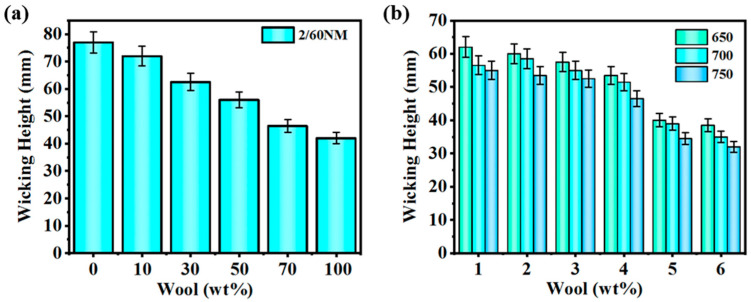
Wicking height of blended yarns with different wool contents: (**a**) 2/60 NM yarn and (**b**) 1/60 NM yarn blended with 650, 700, and 750 T/m.

The wicking performance analysis of wool/PA56 blended yarn is shown in Figure 4. Figure 4a shows the wicking performance of 2/60 NM yarn. As the wool content of the yarn increases, the wicking performance of the blended yarn also increases. The wicking height of pure PA56 yarn is 77.0 mm, while that of 100 wt% wool yarn is 42.0 mm. The reason for this is that the hygroscopic performance of PA56 is better than that of wool fiber. The difference in moisture absorption performance between wool and PA56 fiber is due to their material structure and moisture absorption mechanism. Although wool has good hygroscopic properties, it mainly absorbs and stores moisture through the scales on the fiber surface and the internal channel structure. In contrast, the hygroscopic performance of PA56 fiber is more prominent, which may be related to its unique molecular structure. PA56 fiber is polymerized from biobased pentanediamine and petroleum-based adipic acid. Its molecular chain structure contains a large number of hydrophilic carbonyl groups and amino groups. The presence of these groups increases the absorption and the internal migration of PA56 fiber to water ability, thereby enhancing its hygroscopic properties and thereby improving the wicking properties of the blended yarn. As shown in Figure 4b, the wicking height of blended yarns with the same wool content and different twists gradually decreases as the twist increases. The amount of twist directly affects the capillary effect formed between fibers in the yarn. Generally speaking, yarns with smaller twists have larger gaps between fibers and form longer capillaries, so the wicking height is also higher. As the twist increases, the fibers will be too close together and the capillaries may be blocked, resulting in reduced wicking performance. At the same time, the increase in twist means an increase in the positive pressure between fibers, which increases the friction between fibers and helps maintain the stability of the fibers in the fabric but may also hinder the transmission of liquid, thereby affecting the wicking performance.

### 3.3. Boiling Water Shrinkage Analysis of Yarns

The boiling water shrinkage of wool/PA56 blended yarns is shown in Table 1. The reason for the shrinkage of pure PA56 yarn through heat is, on the one hand, due to the straightened orientation state of the macromolecules in the amorphous region that was energized by the heat and the de-orientation that occurs to re-form the crimped state. On the other hand, it was because the mid-crystalline region caused by the drafting in the process of PA56 staple fibers is also oriented along the axial orientation via the action of the external force during the processing of the fiber preparation. The fiber was de-orientated in the crystalline region after the heat but the degree of the de-orientation was smaller, albeit to a lesser extent. With the addition of wool fibers, the heat shrinkage of PA56 yarns was significantly improved, with 2/60 NM, 70 T/m PA56 decreasing from 4.8% shrinkage to 0%. The 1/60 NM, 650, 700, and 750 T/m pure PA56 yarns with 0% wool content had a boiling water shrinkage of 4.0, 5.2, and 2.0%, respectively, while the 10 wt% wool/PA56 blended yarns all had 0% boiling water shrinkage. Wool/PA56 blended yarn improves the boiling shrinkage of PA56 yarn due to the following reasons: Firstly, wool fibers have high hygroscopicity and can absorb moisture in the air. In a humid environment, the fibers expand after absorbing moisture, which increases the contact area between wool and nylon fibers, making the interaction between the fibers enhanced. This interaction can hinder the shrinkage and boiling shrinkage of nylon fibers. Secondly, wool fibers have good temperature regulation properties, which can change their structure and morphology at different temperatures. At high temperatures, wool fibers are deformed and fused, forming physical cross-links with the nylon fibers and, thus, increasing the stability of the nylon fibers and preventing their boiling shrinkage. Third, there are structural differences between the fibers: wool fibers have a rough outer surface while nylon fibers are smoother. This structural difference leads to physical adsorption between wool fibers and nylon fibers, which increases the interaction between fibers and prevents the shortening and boiling shrinkage of nylon fibers.

### 3.4. Microscopic Morphology Analysis of Yarns

**Figure 5:**
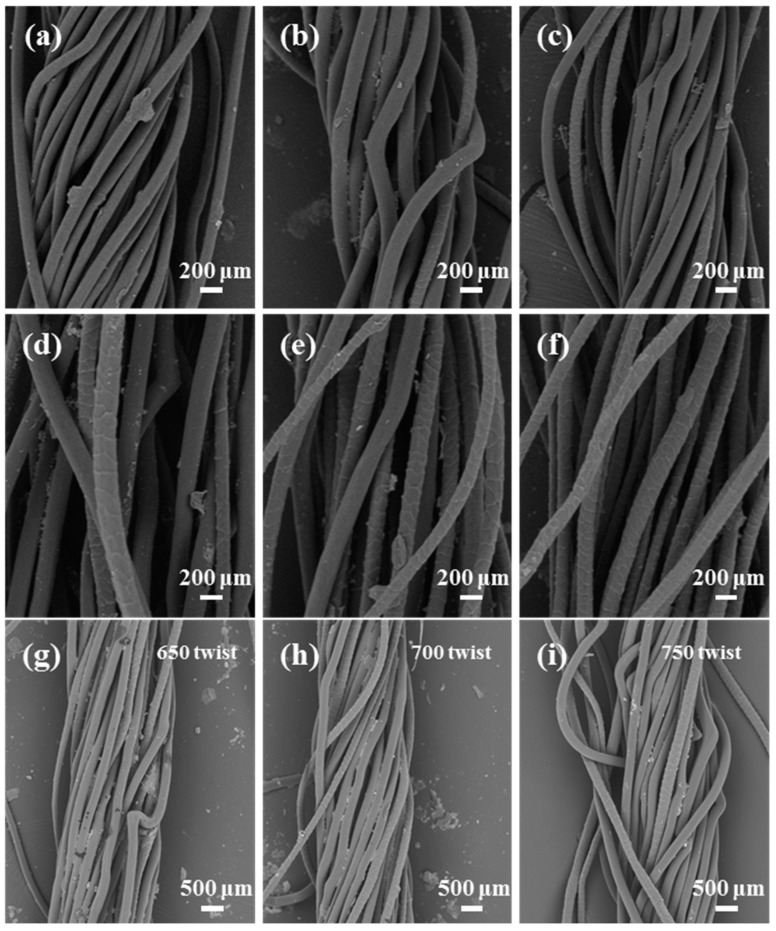
SEM images of blended yarns with different wool contents: (**a**) PA56 yarn, (**b**) 10 wt% wool/PA56 blended yarn, (**c**) 30 wt% wool/PA56 blended yarn, (**d**) 50 wt% wool/PA56 blended yarn, (**e**) 70 wt% wool/PA56 blended yarn, and (**f**) 100 wt% wool yarn, (**g**–**i**) 650, 700, 750 twist of yarn, respectively.

Figure 5a–f represent the SEM images of blended yarns with wool contents of 0, 10, 30, 50, 70, and 100 wt%, respectively. As shown, wool and PA56 fibers can be clearly distinguished. It can be clearly seen that the wool fiber surface has a scale structure and the fiber diameter is different, while the PA56 fiber surface is smooth and the fiber diameter is almost the same. At the same time, as the proportion of wool increases, the yarn increasingly contains wool fibers, which is clearly noticeable. As shown in Figure 5g–i, with the increase in twist, the twist angle of single fibers in the yarn also becomes larger, which makes the fibers in the yarn entangled and interwoven with each other more closely, thus increasing the structural tightness and internal friction of the yarn, which makes the strength of the yarn improved to some extent. With the increase in wool content, wool/PA56 blended yarn can be clearly seen on the surface of the wool fibers; wool fibers have good abrasion resistance, a soft feel, strong moisture absorption, and thermal insulation properties. The same can be said of the abrasion resistance and moisture absorption properties of the high-strength biobased PA56 combination in clothing, work textiles, transport interior textiles, and so on: this has a broad prospect.

### 3.5. Strength Analysis of Yarns

**Figure 6:**
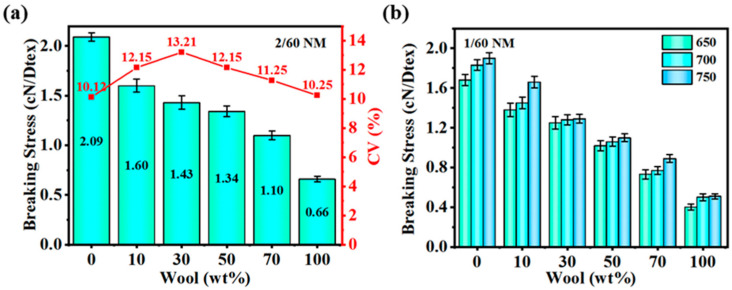
Strength plots of blended yarns with different wool contents: (**a**) 2/60 NM, 700 T/m blended yarn and (**b**) 1/60 NM yarns of 650, 700, 750 T/m blended yarns.

The strength of yarn is one of the important indices in the physical properties of yarn, and it is also a key factor affecting the practical application of the yarn. Figure 6a shows the strength and cv graphs of 2/60 NM, 700 T/m with different wool contents, from which it is obvious that the strength of pure PA56 yarn is 2.09 cN/Dtex with a CV value of 10.12%, and with the increase in wool content, the strength of the blended yarn decreases sharply at first, followed by a slow decrease, and then a rapid decrease. When the wool content was 10 wt%, the strength of the blended yarn was 1.60 cN/Dtex, with a 23.44% decrease in strength. When the wool content was 30 wt%, the strength of the blended yarn was 1.43 cN/Dtex, with a 10.63% decrease in strength. When the wool content was 50 wt%, the strength of the blended yarn was 1.34 cN/Dtex, and there was a 6.3% decrease in strength; however, when the wool content was 70 wt%, the strength of the blended yarn was 1.10 cN/Dtex, indicating an 18% decrease in strength. The reason for this is that wool fibers may have shorter lengths and more lint than PA56 fibers. Short fiber lengths and lint accumulation can cause fibers to slip more easily in the yarn, reducing the strength of the yarn. Differences in chemical structure and physical properties between wool and nylon fibers may result in different cohesion between the fibers. As the wool content increases, the cohesion between the fibers may weaken, affecting the overall strength of the yarn. Figure 6b shows the strength of the blended yarns of 1/60 NM yarns at 650, 700, and 750 T/m. The trend in the strength of the blended yarns with increasing wool content is consistent with Figure 6a, with the strength of the blended yarn at 700 T/m being 1.06 cN/Dtex when the wool content is 50 wt%. The reason that the strength of the yarn increases as the twist increases is that as the twist increases, the fibers in the yarn become denser in the yarn by increasing the contact area between the fibers as they become entangled with each other through the increased twist. This increased compactness of the fibers enhances the overall strength of the yarn. At the same time, increasing the twist increases the friction between the fibers within the yarn. When the friction between the fibers increases, it is more difficult for the fibers to slide and detach from each other when a force is applied, thus increasing the tensile strength of the yarn.

### 3.6. Analysis of Yarn Elongation at Break

**Figure 7:**
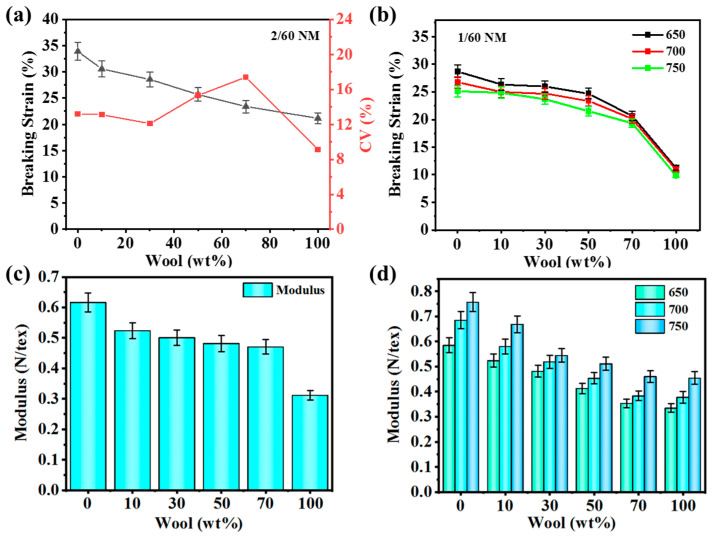
Elongation and modulus of blended yarns with different wool contents: (**a**) elongation of 2/60 NM, 700 T/m blended yarn; (**b**) elongation of 1/60 NM yarns of 650, 700, 750 T/m blended yarns; (**c**) modulus of 2/60 NM, 700 T/m blended yarn; (**d**) modulus of 1/60 NM yarns of 650, 700, 750 T/m blended yarns.

As shown in Figure 7a, in the blended yarn of the 2/60 NM, 700 T/m specification, the elongation at break of pure PA56 yarn is 33.92%, and with the increase in wool content, the elongation at break of wool/PA56 blended yarn decreases, and the elongation at break of 100% wool yarn is 21.15%, which is caused by this phenomenon, because of the low elongation of the wool yarn itself. Figure 7b shows the blended yarns of 1/60 NM yarns of 650, 700, and 750 T/m. The trend in elongation at break with the increase in wool content is similar to that of Figure 7a, and the elongation at break of 700 T/m blended yarn is 23.39% when the wool content is 50 wt%. The elongation at break of the yarn showed a decreasing trend with the increase in twist, and the fibers were stretched and oriented during the twisting process, resulting in an increase in the strength of the yarn and a decrease in the elongation at break.

Figure 7c,d are bar charts of the yarn modulus. It can be seen from the figures that as the wool content increases, the modulus of blended yarn decreases, which is due to the softness characteristics of wool yarn itself. The results indicate that wool fibers can effectively improve the softness of nylon yarn. As the twist increases, the modulus of the yarn also increases, and its softness decreases. Yarns with lower twist are softer because the friction between fibers is smaller, making them more prone to bending and deformation. As the twist increases, the friction between fibers increases, and the yarn becomes tighter and harder, while the softness decreases.

### 3.7. Stem Analysis of Yarns

**Figure 8:**
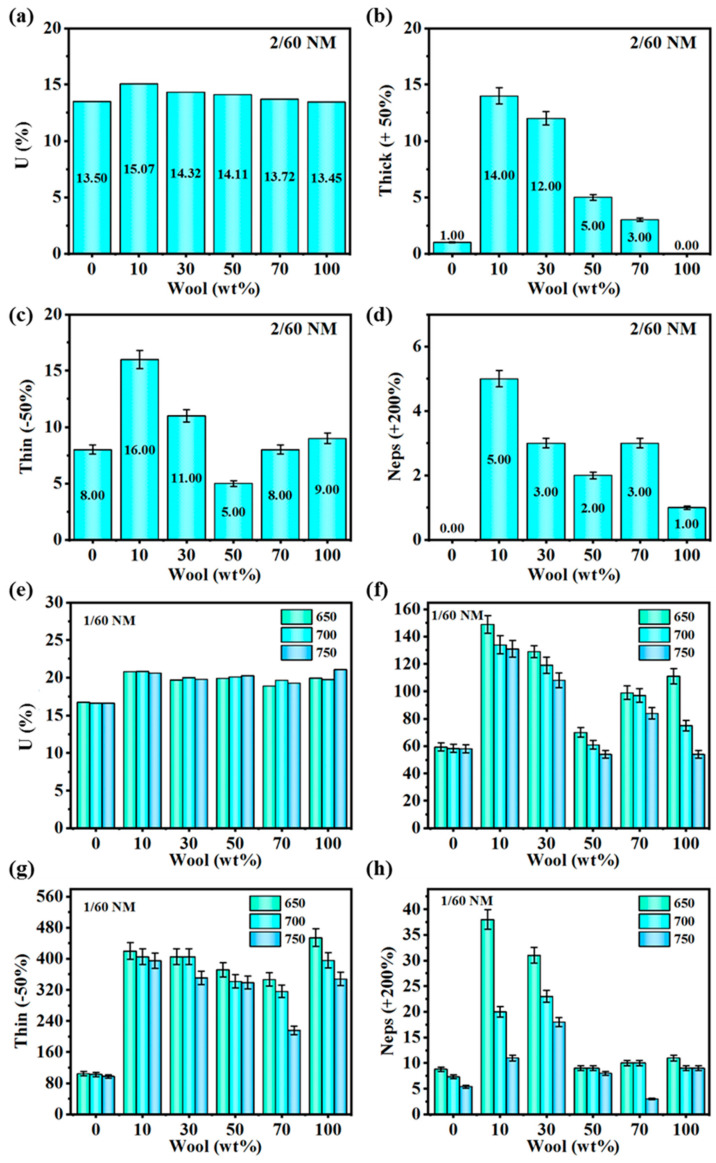
(**a**) Uster stem of 2/60 NM, 700 T/m blended yarn; (**b**) thick knots of 2/60 NM, 700 T/m blended yarn (+50%); (**c**) details of 2/60 NM, 700 T/m blended yarn (−50%); (**d**) cotton knots of 2/60 NM, 700 T/m blended yarn (+200%); (**e**) 1/60 NM blended yarn with UST stem; (**f**) 1/60 NM blended yarn with thick knots (+50%); (**g**) 1/60 NM blended yarn with details (−50%); (**h**) 1/60 NM blended yarn with cotton knots (+200%).

Strip dryness has a great influence on the strength and uniformity of the yarn, the stability of textile processing, and the quality and feel of the fabric; once the composite yarn has details or thick knots and other yarn unevenness, the apparent performance of the yarn, as well as the weaving performance of the fabric in the later stage of the use of the fabric, will be affected. As shown in Figure 8a’s 2/60 NM, 700 T/m blended yarn Uster stem graph and as shown in Figure 8e’s 1/60 NM blended yarn Uster stem graph, it can be clearly seen that with the increase in wool content, CV value increases first, and then the wool content of the blended yarn is increased; the CV values reached a maximum when the wool content was 10 wt%, 15.07% in 2/60 NM, 700 T/m blended yarn and 20.83% in 1/60 NM, 700 T/m blended yarn. As the wool content continued to increase, the CV values began to decrease; when the wool content in the yarn was 50%, the CV value was 14.11% in 2/60 NM, 700 T/m blended yarn and when the wool content in the yarn was 100%, the CV value was 13.45% in 2/60 NM, 700 T/m blended yarn and 19.76% in 1/60 NM, 700 T/m blended yarn. The reason why the dryness of the blended yarn of 2/60 NM is smaller than that of 1/60 NM is that the blended yarn of 2/60 NM is made by twisting two strands of yarn, which can make the fibers more tightly combined in the yarn, the fiber lengths and distributions of the two strands of yarn will match with each other, and the dryness of the two strands of yarn may have a counteracting effect during the twisting process, which will result in the overall dryness value to be smaller than that of the blended yarn of 1/60 NM in terms of the CV value.

In Figure 8b–d, the histograms of thick knots (+50%), details (−50%), and cotton knots (+200%) of 2/60 NM, 700 T/m blended yarns are shown, respectively, and it can be analyzed that the values of thick knots (+50%), details (−50%), and cotton knots (+200%) of 2/60 NM, 700 T/m blended yarns firstly increase and then decrease with the increase in wool content. The values of thick knots (+50%), details (−50%), and cotton knots (+200%) of the blended yarns with wool content of 10 wt% reached the maximum values of 14.0, 16.0, and 5.0, respectively. The values of thick knots (+50%), details (−50%), and cotton knots (+200%) of the blended yarns decreased as the wool content continued to increase. At a wool content of 50 wt%, the values of detail (−50%) and cotton knots (+200%) reached 5.0 and 2.0. Wool fibers are shorter than PA56 staple fibers, which increases fiber entanglement and knotting during spinning. PA56 long fibers are better able to maintain continuity during spinning, reducing breakage and lint, thereby reducing the number of neps in the yarn. However, as the wool content increases, the fiber distribution in the yarn is more even, and the cohesion between the fibers is enhanced. This helps improve the overall strength and uniformity of the yarn.

As shown in Figure 8f–h for 1/60 NM, respectively, the bar graphs of thick knots (+50%), details (−50%), and cotton knots (+200%) of blended yarns, the overall trend in the blended yarns with increasing wool content is the same as that of Figure 8b,c for 2/60 NM, respectively, and the bar graphs of thick knots (+50%), details (−50%), and cotton knots (+200%) of 700 T/m blended yarns. The trends were the same. The values of thick knots (+50%), details (−50%), and cotton knots (+200%) decreased with increasing twist, which is attributed to the fact that high twist can make the fibers in the yarn more tightly bonded together, reduce fiber loosening and breaking, and lead to the better uniformity of the yarn. Better uniformity reduces the creation of larger differences in thickness and makes the yarn look more detailed.

**Figure 9:**
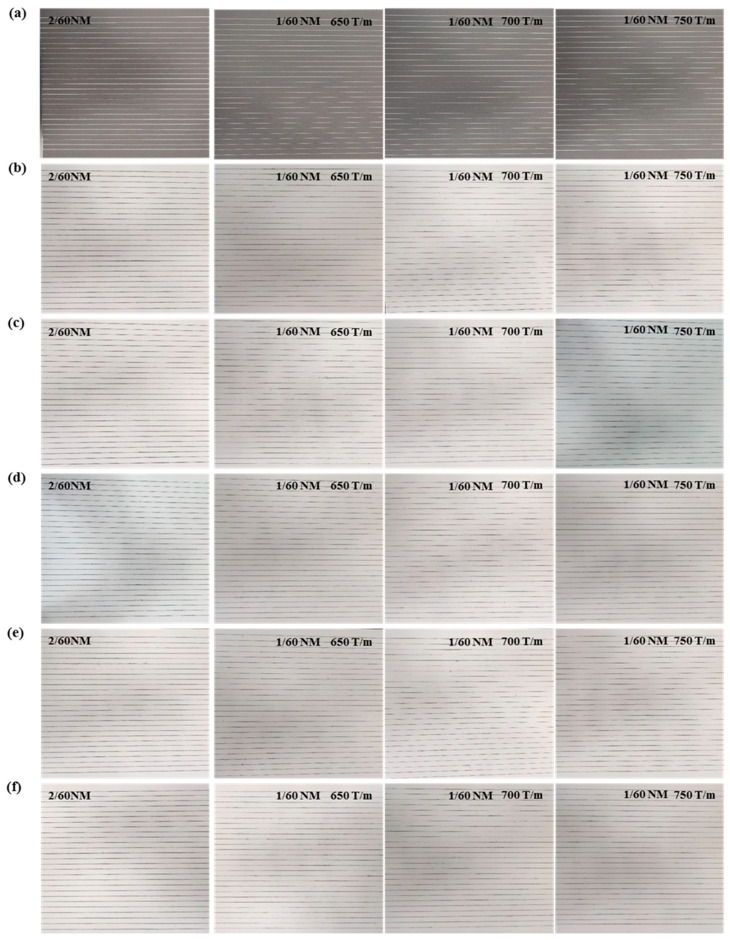
Blackboard diagram images of blended yarns with different wool contents: (**a**) PA56 yarn, (**b**) 10 wt% wool/PA56 blended yarn, (**c**) 30 wt% wool/PA56 blended yarn, (**d**) 50 wt% wool/PA56 blended yarn, (**e**) 70 wt% wool/PA56 blended yarn, and (**f**) 100 wt% wool yarn.

As shown in Figure 9, the blackboard diagram of blended wool/PA56 yarn, the analysis showed that the yarn backbone performance is not only related to the proportion of wool in the blended yarn but is also affected by the twist of the blended yarn. The analysis showed that when the wool content of the blended yarn is 50 wt%, the evenness performance is better. As the twist increases, the evenness performance first becomes better and then decreases. When the twist is 700, the evenness performance of the yarn is better. The reason was that high twist can make the short fibers of the blended yarn more closely combined, thereby improving the yarn’s evenness properties. However, too high a twist would cause stress concentration inside the yarn, which would create weak points in the yarn and affect the evenness.

### 3.8. Hairiness Analysis of Yarns

**Figure 10:**
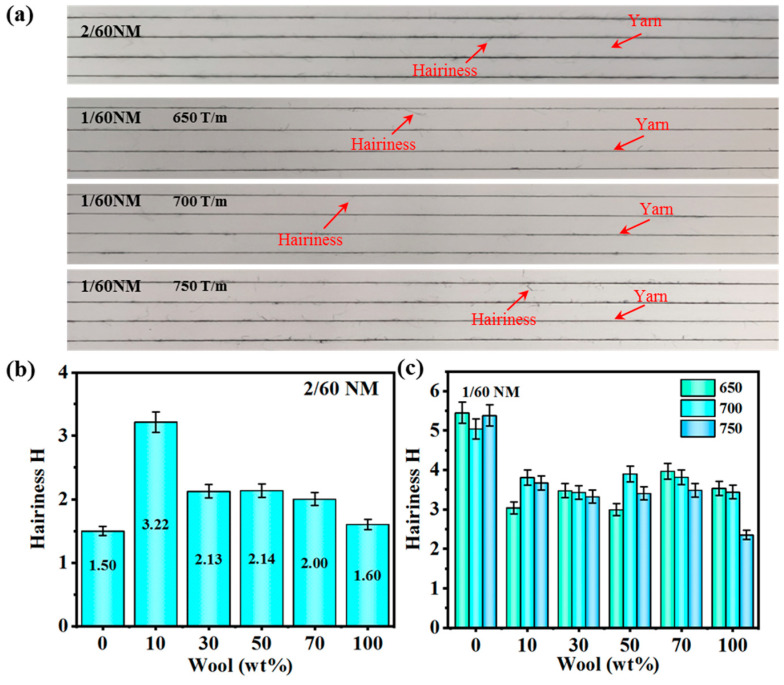
(**a**) Yarn diagram; (**b**) hairiness of 2/60 NM, 700 T/m blended yarn; (**c**) hairiness of 1/60 NM blended yarns.

Figure 10a shows the hairiness photos of 2/60 NM yarn and with different twists of 1/60 NM yarns. As shown in Figure 10b, the hairiness graph of 2/60 NM, 700 T/m blended yarn, the hairiness value increases and then decreases with the increase in wool content. When the wool content is 0 wt%, the hairiness H-value of pure PA56 yarn is 1.50, when the wool content is 10 wt%, the hairiness H-value of blended yarn reaches a maximum of 3.22, and the hairiness H-value of blended yarn decreases as the wool content continues to increase; the hairiness H-value of 50 wt% wool yarn is 2.14 and the hairiness H-value of pure wool yarn is 1.60.

Shown in Figure 10c is the hairiness graph of 1/60 NM blended yarn; as the wool content increases, the hairiness value first increases and then decreases. The overall trend is the same as that in Figure 10b; as the twist increases, the yarn fibers are more tightly bonded to each other, which makes the number of fibers protruding from the yarn smaller, resulting in a decrease in the H-value of hairiness. A comprehensive comparison of 2/60 NM and 1/60 NM yarns with the same wool content shows that the 2/60 NM yarn has a little better hairiness H-value. The reason for this is that the two strands of yarn are combined together via twisting during the spinning and weaving process, and the fiber length and distribution of the two strands of yarn will match each other, making the fiber length distribution of the two strands of yarn relatively uniform, which leads to a reduction in the overall hairiness H-value.

### 3.9. Friction Performance Analysis of Yarn

**Figure 11:**
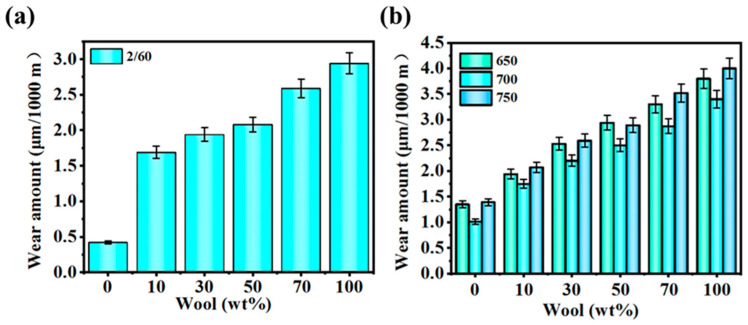
(**a**) Wear amount of 2/60 NM, 700 T/m blended yarns; (**b**) wear amount of 1/60 NM blended yarns.

The wear resistance analysis of the yarn is shown in Figure 11. In the yarn specification of 2/60 NM, the abrasion loss of pure PA56 yarn is 0.42 μm/1000 m and that of the 50% wool blended yarn is 2.08 μm/1000 m. The abrasion loss of the 100% wool blended yarn is 2.94 μm/1000 m. In the yarn specification of 1/60 NM, the abrasion loss of yarn with a twist of 700 generally tends to be lower. The test results show that with the increase in yarn twist, the wear resistance of the yarn first increases and then decreases. The reason is that with the increase in yarn twist, the fibers in the yarn are more tightly held together, increasing the strength and wear resistance of the yarn. However, when the twist reaches a maximum value, if the twist continues to increase, the wear resistance will gradually decrease. This is because excessive twist can cause the yarn to become stiff and less prone to flattening, resulting in a decrease in contact area during friction and an increase in local stress, making the yarn more susceptible to damage. In addition, when the twist is too high, the stress attached to the fibers increases, and the fibers lack appropriate movement space in the yarn, which is also detrimental to wear resistance. PA56 has better wear resistance than wool; therefore, as the wool content in the yarn increases, the corresponding wear amount also increases.

## 4. Conclusions

In this paper, blended yarns of wool and biobased PA56 with a green non-polluting nature, energy savings, high strength, abrasion resistance, high elasticity, and good hand comfort were prepared.

It was shown that the strength of the blended yarn decreased with the increase in wool fiber content. When the twist rate was 700, the wool content was 50 wt% and the PA56 content was 50 wt%; the strength of the yarn in a 2/60 NM size reached 1.34 cN/Dtex, and the elongation at break reached 25.71%.

The 2/60 NM blended yarn with 50% wool and 50% PA56 prepared in this study has a good stem (the CV value of the Ulster strip is 14.11%), hairiness (the hairiness H-value is 2.14), and wear resistance (the wear amount is 2.08 μm/1000 m); it has a wide prospect in the field of textiles for apparel and automobile interiors.

## Figures and Tables

**Table 1:** Boiling water shrinkage of yarns with different wool contents.

Public Branch (NM)	Twist (T/m)	Wool					
		0 wt% (%)	10 wt% (%)	30 wt% (%)	50 wt% (%)	70 wt% (%)	100 wt% (%)
2/60	700	4.8	0	0	0	0	0
	650	4.0	0	0	0	0	0
1/60	700	5.2	0	0	0	0	0
	750	2.0	0	0	0	0	0

## Data Availability

Data are contained within the article.
